# Technical note: Generation of a Cerenkov scatter function convolution kernel for a primary proton beam

**DOI:** 10.1002/acm2.13083

**Published:** 2020-10-30

**Authors:** Steven A. Thompson

**Affiliations:** ^1^ Department of Medical Physics University of Florida Gainesville FL USA

**Keywords:** Cerenkov radiation, Cerenkov scatter function, convolution, proton therapy

## Abstract

**Purpose:**

To generate a Cerenkov scatter function (CSF) for a primary proton beam and to study the dependence of the CSF on the irradiated medium.

**Materials and Methods:**

The MCNP 6.2 code was used to generate the CSF. The CSF was calculated for light‐pigmented, medium‐pigmented, and dark‐pigmented stratified skin, as well as for a homogeneous optical phantom, which mimics the optical properties of human tissue. CSFs were generated by binning all of the Cerenkov photons which escape the back end (end opposite beam incidence) of a 20 × 20 × 20 cm phantom. A 4 × 4 cm, 500 × 500 bin grid was used to create a histogram of the Cerenkov photon flux on the simulated medium’s back surface (surface opposite beam incidence). A triple Gaussian was then used to fit the data.

**Results:**

From the triple Gaussian fit, the coefficients of the CSF for the four phantom materials was generated. The individual CSF fit coefficient errors, with respect to the Gaussian representation, were found to be between 0.92% and 4.11%. The R^2^ value for the fit was calculated to be 0.99. The phantom material was found to have a significant effect (63% difference between materials) on the CSF amplitude and full width at half maximum (195% difference between materials). The difference in these parameters for the three skin pigments was found to be small.

**Conclusions:**

The CSF was obtained for a proton beam using the MCNP 6.2 code for a phantom constructed of light, medium, and dark stratified human skin, as well as for an optical phantom. The CSFs were then fit with a triple‐Gaussian function. The coefficients can be used to generate a radially symmetric CSF, which can then be used to deconvolve a measured Cerenkov image to obtain the dose distribution.

## 1 INTRODUCTION

Proton therapy (PT) is a form of radiation therapy which is rapidly increasing in use. PT has the advantage of significantly reduced toxicity to non‐malignant tissue due to the absence of exit dose, while providing focused high linear energy transfer radiation to the tumor target. However, real‐time *in vivo* image guidance, which provides in situ information on the position of the tumor target within the beam aperture, is hindered by the finite range of the protons.

There has been increasing interest in using Cerenkov emissions for quality assurance and *in vivo* dosimetry for both electron^1^ and photon^2^ therapy. Although a primary proton beam does not have sufficient energy to generate Cerenkov emissions directly (about 482 MeV is required to produce Cerenkov emissions in water^3^), it has been demonstrated that there are two secondary mechanisms by which such emissions may occur indirectly: (1) a fast linear component from fast electrons liberated by prompt gamma(99.13%) and neutron (0.87%) emission; and (2) a slow non‐linear component arising from the decay of radioactive positron emitters.^4^ The present work seeks to develop an *in vivo* dose measurement method using Cerenkov radiation produced by secondary electrons and positrons within the patient volume. This could be helpful for evaluating the accuracy of the prescribed radiation treatment, monitoring hot spots of dose deposition, and improving the therapeutic outcomes of PT due to the ability to accurately monitor dose in real time. However, there is no method that can perform this type of dosimetry in real time with sufficient accuracy.

Proton therapy beams produce electrons and positrons that are responsible for dose deposition in a medium. Above a certain energy threshold, these charged particles will be superluminal within the medium and emit Cerenkov photons along their path. The Frank‐Tamm formula gives the amount and spectrum of Cerenkov photons emitted.^5^ The light‐to‐dose relationship has been approached as a deconvolution problem.^6^ Most Cerenkov photons should be emitted in locations proximal to radiation dose deposition, since the proton beam imparts energy due to direct ionization, and therefore deconvolution by a light‐transfer function can be used to isolate the corresponding regions of dose.

This approach has been used for photon and electron beams^7^, ^8^, but not for PT beams. In these studies, a Cerenkov scatter function (CSF) was introduced, which represents the collection of all scattered Cerenkov photons that are emitted from the surface of a medium irradiated by a pencil beam of high‐energy radiation. The CSF relates Cerenkov photon images taken during treatment to the surface fluence of primary radiation particles through a deconvolution relationship. The beam fluence can then be used to determine the superficial dose deposition using the relationship between beam fluence and the dose scatter function (DSF).^9^

To date, no CSF has been produced for a primary proton beam. In this work, a CSF is generated for a proton therapy beam for four different tissue equivalent phantom material compositions. The MCNP 6.2 code is used to simulate the interactions in the phantom.

## 2 THEORY

### 2.A Cerenkov radiation

Cerenkov radiation is electromagnetic radiation which is emitted by charged particles passing through a dielectric material at a speed in excess of the phase velocity of light in that material. When a charged particle moves inside a dielectric material, it excites molecules to higher energy levels and excited states. Upon returning back to their ground state, the molecules re‐emit some photons in the form of electromagnetic radiation. According to Huygens principle, the emitted waves move out spherically at the phase velocity of the medium. If the particle motion is slow, the radiated waves bunch up slightly in the direction of motion, but they do not cross. However, if the particle moves faster than the light speed, the emitted waves add up constructively leading to a coherent radiation at an angle with respect to the particle direction, known as Cherenkov radiation. The result of this effect is a cone of photon emission in the direction of particle motion.

### 2.B Cerenkov scatter function

**FIG. 1:**
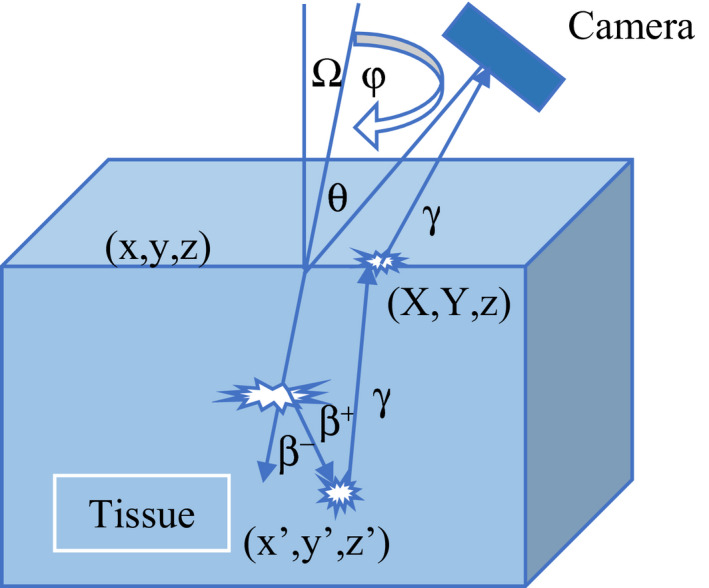
Coordinate system of the Cerenkov radiation creation process and subsequent detection. The 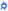 symbols represent interaction points.

Figure 1 shows the physical processes and coordinate system used in the mathematical formulation that follows.

A modulated proton beam incident on a tissue volume in the Z‐direction is considered. A system capable of imaging Cerenkov photons is placed at some angle θ relative to the beam. The proton beam produces secondary electrons and positrons (mostly from ^15^O) which are responsible for both energy deposition and the creation of Cerenkov photons within the medium. Cerenkov photons created within the tissue volume will undergo scattering and absorption. Some of the Cerenkov photons will be absorbed, but a fraction (about 1%)^9^ are scattered toward the surface and escape from the tissue volume, which can then end up being detected in the imaging system. The imaging system can be used to collect the escaped Cerenkov photons and take a planar image of the surface of the medium.

The following convolution equation represents the relationship between the surface flux of the incident proton beam entering the tissue volume and the intensity of the Cerenkov photons reaching the imaging system^6^:

(1) I(θ)=J(Ω)∗CSF(Ω,θ)∗PSF

where I is the Cerenkov photon intensity at the imaging system, J is the surface flux of the incident proton beam, PSF is the point spread function representing the transport of Cerenkov photons from the surface of the medium, (X, Y, z) in Figure 1, to the imaging system, and CSF is the Cerenkov scatter function, which represents the Cerenkov photons which escape the tissue volume from a pencil beam incident on the surface. Equation 1 shows that the image formed by the scattered Cerenkov photons is a convolution of the surface flux J by the CSF and PSF convolution kernels. ^6^

The CSF can be written as a convolution of the dose scatter function (DSF) kernel and the Cerenkov dose scatter function (CDSF) kernel as follows:

(2) $$\text{CSF}\left(\Omega ,\theta \right)=\text{CDSF}\left(\Omega ,\theta ,d_{0}\right)*\text{DSF}\left(\Omega ,\theta ,d_{0}\right)$$

The CDSF kernel represents the production of Cerenkov photons by the secondary electrons and positrons from the incident proton beam and the transport of these Cerenkov photons toward the surface of the tissue volume. Equation 2 indicates that the CDSF can be found by deconvolving the CSF with the DSF.^9^

The dose deposited in a plane at depth d_0_ can be written as:

(3) $$D\left(d_{0}\right)=j\left(\Omega ,E\right)*\text{DSF}\left(\Omega ,\theta ,d_{0}\right),$$

where E is the incident particle energy.

Inserting Equation 2 into Equation 1, and using Equation 3 for the planar dose distribution D, I can be expressed as a convolution of CDSF and D as follows:

(4) $$I\left(\theta \right)=\text{CDSF}\left(\Omega ,\theta ,d_{0}\right)*D\left(d_{0}\right)$$

The objective now is to determine the 2D dose distribution at depth d_0_ in the tissue volume; the problem then becomes one of deconvolution of the Cerenkov photon intensity I, which is captured by the camera, with the CDSF, which can be found by deconvolving with the CSF produced herein and the DSF which will be calculated in a later work. The result is the dose profile at depth d_0_.

### 2.C Mathematical assumptions

The convolution statements in Equations (1), (2), (3), (4) require a few mathematical assumptions. If it is assumed for modeling simplicity that an ideal imaging system is being considered (100% absorption of escaped Cerenkov photons), the PSF could be represented by a Dirac‐delta function, which would allow the PSF term in Equation 1 to be ignored. Also, in order to justify the convolution formulations of Equations (1), (2), (3), (4), all of the convolution kernels must be shift invariant. This requirement can be satisfied by requiring the incident proton beam to be normal to the surface of the homogeneous medium. Therefore, a 0^o^ beam angle is assumed in the present work.

## 3 METHODS

### 3.A MCNP Monte Carlo Simulation

The MCNP 6.2^10^ code was used to generate the CSF. The Cerenkov methodology for MCNP is described in detail in Reference 11. The feature is assiduously tested in that work. The CSF was calculated for light‐pigmented, medium‐pigmented, and dark‐pigmented stratified skin, as well as for a homogeneous optical phantom, which mimics the optical properties of human tissue.

The MCNP model consisted of a pencil beam proton radiation source and the phantom being irradiated, with air surrounding. The radiation source was directed at the phantom with a constant beam angle of θ_in_ = 0°, where θ_in_ is the angle of entrance vector defined from the normal surface. As aforementioned, this is to ensure shift invariance. The phantom was modeled as a 20 x 20 x 20 cm^3^ box. The CSF simulations used the spread‐out Bragg peak (SOBP) shown in Table 1 below; these data were taken from the University of Florida Proton Therapy Institute treatment planning computer.

**Table 1:** Proton energies and weightings for spread out Bragg Peak Curve.

Proton energy incident on phantom (MeV)	Weighting (relative to maximum energy)
147.077007	1
144.47285	0.2913
141.8320352	0.2829
139.095367	0.1957
136.37493	0.1854
133.6120477	0.1240
131.7054986	0.1187
128.8660584	0.1354
125.9776021	0.1063
122.7212308	0.1164
119.7196471	0.0872
116.3956217	0.0965
113.266237	0.0738
110.0688085	0.084
106.6573136	0.071
103.3041808	0.0838
99.86505505	0.0542
96.33220172	0.0615
92.69662631	0.0565
88.54178403	0.0585

The CSF is created by the transport of Cerenkov photons created by a pencil beam of ionizing radiation incident on a dielectric material. For PT, a high‐energy proton beam is the primary radiation beam. The primary proton beam produces secondary electrons and positrons, which can exceed the speed of light in the medium, producing Cerenkov photons. Most of these photons will be absorbed in the tissue, but about 1% travel toward the surface and escape the irradiated medium.^9^ A Cerenkov detector‐imaging system can be used to image these escaped photons.

The initial position and direction at the time of the Cerenkov photon creation and the final position and final direction at which the Cerenkov photon escapes the phantom were recorded. The CSF was calculated by scoring the flux of the Cerenkov photons which escaped from the phantom surface. The positions of the photons leaving the surface were scored in a grid of 0.2 x 0.2 mm^2^ pixels.

### 3.B Material properties

Four different phantom materials were used in this study: light‐pigmented, medium‐pigmented, and darkened‐pigmented stratified skin and an optical phantom material. The stratified skin models were included to allow the effect of material properties, in particular the melanin content, on the CSF to be studied. The compositions for all four materials were taken from the National Institute of Standards and Technology soft tissue library.^12^

### 3.C Generation of Cerenkov scatter function

CSFs were generated by binning all of the Cerenkov photons which escape the back end of the phantom (end opposite beam incidence). A 4 x 4 cm^2^, 500 x 500 bin grid was used to create a histogram of the Cerenkov photon flux on the simulated medium surface. The histogram was sampled along a line from r = −10 to 10 cm using spoke‐sampling which was centered at the origin of the pencil beam on the medium surface. The sampling angle, θ_s_, was defined on the medium surface as the rotation from the Y‐axis about the Z‐axis. Sampling angles of 0‐180 degrees in one‐degree increments were used. A triple‐Gaussian distribution was used to fit the mean of the data:

(5) $${\text{CSF}}_{\text{mean}}\left(\alpha ,\beta ,\gamma ,\delta ,\varepsilon ,\zeta \right)=\alpha e^{\frac{‐{\left(r\right)}^{2}}{\beta }}+\gamma e^{\frac{‐{\left(r\right)}^{2}}{\delta }}+\varepsilon e^{\frac{‐{\left(r\right)}^{2}}{\zeta }}$$

**FIG. 2:**
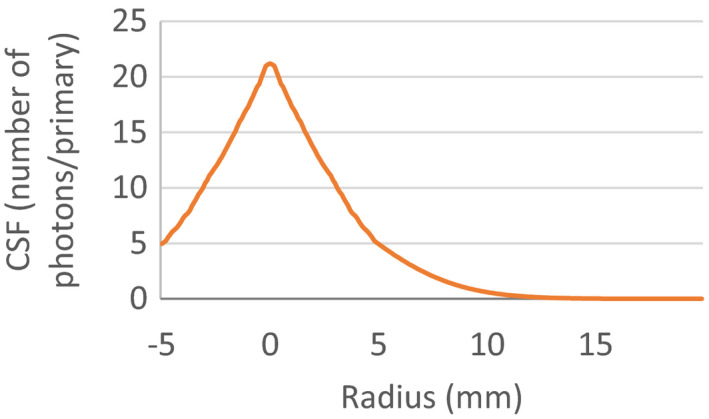
CSF Gaussian Fit for Optical Phantom Model.

CSF_mean_ is the mean of the CSF cross‐sections over the sampling angle θs. The parameter r is the radial distance from the entrance of the proton pencil beam on the optical phantom surface. The parameters α, β, γ, δ, ε, and ζ are fit coefficients, generated using the Mathworks MATLAB fitnlm function; they were scaled by the number of primary protons incident on the optical phantom. The fitnlm function also produces the error in the fit coefficients. The triple‐Gaussian fit was chose due to it having the best goodness of fit (R^2^) value of the fits tested, as well as having the lowest error in the fit coefficients. Single‐Gaussian and double‐Gaussian fits were also tested but yielded lower R2 values and larger errors in the fit coefficients. The error in CSF_mean_ was found as the standard deviation at each radial point from the CSF cross sections. The large number of histories and the incorporated variance reduction ensured that the error at each point on CSF_mean_ was <1% of the maximum value for each CSF. The Gaussian fit of the CSF data is shown in Figure 2 below. The full width at half maximum (FWHM) and the amplitude was determined for each CSF.

## 4 RESULTS

The coefficients of the CSF for the four phantom materials are shown in Table 2 below. The individual coefficient errors were found to be between 0.92% and 4.11%, indicating that the Gaussian fitting using Equation 6 was appropriate for the data. The R^2^ value was calculated to be 0.99.

**Table 2:** Gaussian fit coefficients for CSF.

	α(γs/primary)	β(mm)	γ(γs/primary)	δ(mm)	ε(γs/primary)	ζ(mm)
Optical Phantom	6.14 × 10^‐5^	2.16	1.38 × 10^‐4^	4.81	5.93 × 10^‐5^	8.42
Light Skin	7.92 × 10^‐5^	1.11	4.66 × 10^‐5^	3.92	1.81 × 10^‐6^	4.26
Medium Skin	7.49 × 10^‐5^	1.11	4.47 × 10^‐5^	3.92	1.48 × 10^‐6^	4.26
Dark Skin	5.30 × 10^‐5^	1.11	4.19 × 10^‐5^	3.90	1.33 × 10^‐6^	4.27
% error range	1.81‐4.01	0.92‐2.19	1.46‐3.79	1.12‐2.01	3.00‐4.11	1.33‐1.59

Table 3 below shows the width and amplitude of the CSFs. The amplitudes were normalized to the number of primary protons incident on the optical phantom. As can be observed from the table, due to the increased absorption due to melanin, the CSF amplitude decreased with melanin content.

**Table 3:** Amplitude and FWHM for the CSF.

	Amplitude (photons/primary particle)	FWHM (mm)
Optical Phantom	2.12 × 10^‐4^	5.72
Light Skin	1.30 × 10^‐4^	1.94
Medium Skin	1.22 × 10^‐4^	1.93
Dark Skin	1.09 × 10^‐4^	1.99

## 5 DISCUSSION

In the present work, the CSF was calculated for a proton beam incident on phantoms of light, medium, and dark stratified skin, as well as an optical phantom, which mimics the optical properties of human tissue. The SOBP from Table 1 was used for the energy distribution of said beam.

The material was found to have a significant effect on the CSF amplitude and FWHM. The optical phantom CSF amplitude was found to be 63% greater than the light skin phantom; the FWHM for the optical phantom was found to be 195% greater than the light skin phantom material. The skin pigment was found to have a small impact on the CSF amplitude and almost no effect on the FWHM. The lack of effect on the FWHM is likely due to the fact that the only difference between the three pigments in the melanin content in the epidermis. The other layers of skin have identical scattering and absorption characteristics. As a result, Cerenkov photons have similar behavior underneath the epidermis layer, but have different amounts of absorption between the three skin models for Cerenkov photons leaving the tissue. As a result, the FWHM for the three skin models are similar.

As can be observed from Table 3, the optical phantom produced a CSF amplitude which was greater than the three skin models. In addition, the lateral spread, as represented by the FWHM, was significantly greater for the optical phantom than for the three skin models. This is a result of the significantly smaller absorption cross section for the optical phantom material in comparison with the stratified skin materials. This decreased absorption leads to more Cerenkov photons leaving the phantom surface at a further distance from the pencil beam entrance.

## 6 CONCLUSIONS

In the present work, the CSF was obtained for a proton beam using the MCNP 6.2 code for a phantom constructed of light, medium, and dark stratified human skin, as well as for an optical phantom. The CSFs were then fit with a triple‐Gaussian function using the MATLAB fitnlm function. The coefficients for this fit are provided in Table 2 and can be used to generate a radially symmetric CSF, which can then be used to deconvolve a measured Cerenkov image to obtain the dose distribution if the DSF is known beforehand.

The phantom material was found to have a significant effect on the CSF amplitude and FWHM. The difference in these parameters for the three skin pigments was found to be small, meaning that melanin content has a diminutive effect on the CSF. The % errors for the parameters were found to vary from 0.92% to 4.11%, with an R^2^ value of 0.99.
